# Development of a Model for Predicting Enlarged Prostate Size in Noncastrated Dogs Through B-Mode Ultrasound

**DOI:** 10.1155/vmi/9593213

**Published:** 2025-07-21

**Authors:** Guillermo Cazzuli, Juan Pablo Damián, Paula Pessina, Gonzalo Suárez

**Affiliations:** ^1^Departamento de Clínicas y Hospital Veterinario, Facultad de Veterinaria, Universidad de la República, Montevideo, Uruguay; ^2^Departamento de Biociencias Veterinarias, Facultad de Veterinaria, Universidad de la República, Montevideo, Uruguay; ^3^Núcleo de Bienestar Animal, Facultad de Veterinaria, Universidad de la República, Montevideo, Uruguay

**Keywords:** canine, diagnostic imaging, prostate disorders

## Abstract

The prostate in noncastrated male dogs typically experiences an increase in weight and size as they age, which can result in the development of prostatic disorders. Benign prostatic hyperplasia (BPH) is the most common prostate disorder in these dogs. The purpose of this study was to develop a model that could differentiate between dogs with normal prostate sizes and those with enlarged prostate sizes. To achieve this, the researchers evaluated prostate sizes and volumes estimated by B-mode ultrasound in healthy noncastrated dogs and dogs with BPH, considering factors such as weight, age, aorta diameter, and the presence of intraparenchymal cysts. Two hundred and sixty noncastrated male dogs of various breeds were used, with two hundred and thirty-three dogs in the healthy group and twenty-seven dogs in the BPH group. Data on breed, body weight, and age were collected for each dog, and B-mode ultrasound was performed to obtain prostate variables of length, width, and average height, as well as the presence/absence of intraparenchymal cysts and aorta diameter. All variables presented differences between groups except aorta diameter. All dogs with BPH were older than 6 years (*p* < 0.001), with a higher percentage of prostate cysts (*p* < 0.001) and larger prostate volumes than healthy dogs (81 ± 41 cm^3^ vs. 22 ± 15 cm^3^, *p* < 0.001). The outcomes of this research resulted in the creation of a model that can precisely (98.1%), sensitively (88.9%), and distinctly differentiate between healthy, noncastrated dogs and those with BPH by considering several factors, including body weight, age, aorta diameter, and the existence of intraparenchymal cysts.

## 1. Introduction

In noncastrated male dogs, it is clearly documented that as age increases, there is a gradual increase in the weight and size of the gland [1]. These changes are those that generally predispose to the presentation of prostate diseases [2]. In this sense, benign prostatic hyperplasia (BPH) is the most frequent prostatic disorder in dogs [3, 4] and is defined as an increase in size (hypertrophy) and in the number of epithelial cells of the prostate (hyperplasia) [5]. The hyperplastic prostate tends to develop multiple small cystic spaces [6], which provide an excellent medium for bacterial growth, making BPH the underlying cause of prostatitis, as well as other prostate disorders [7]. Therefore, early diagnosis and treatment of BPH decrease the probability of future associated pathologies [5].

Since most prostate disorders involve enlargement of the gland, evaluating its size is crucial. In this sense, there are numerous ways to assess prostate size, including rectal palpation, radiography, B-mode ultrasound [8–10], computed tomography (CT) [11, 12], and magnetic resonance imaging (MRI) [13]. Of all of them, B-mode ultrasound is the most widely used for the examination of the prostate gland [14] because it is a noninvasive and low-cost technique, allowing visualization of the internal architecture, the margins, and the length, width, and height of the gland [1]. In turn, B-mode ultrasound also allows obtaining ultrasound-guided samples for cytological evaluation, using fine needle aspiration [15]. Previous studies have shown that prostate size (length, width, and height) and volume estimated by B-mode ultrasound are related to age and weight in noncastrated dogs [8, 9], as well as other body parameters, such as the diameter of the abdominal aorta [8, 16]. The dog's species presents great variability in sizes and body weights, and some breeds tend to be thin and other breeds tend to be overweight [17, 18], with the prevalence of overweight and obese dogs being high [19]. Therefore, the use of dog weight as the only body criterion could generate a great variability when estimating prostate size and volume. In this sense, having a model, which includes other body variables, allowing differentiation between normal and enlarged prostates measured by B-mode ultrasound in noncastrated male dogs, could contribute to decision making by veterinary clinicians about prostate health in a noninvasive, accessible, fast, and low-cost way. For this, it is necessary to characterize a large number of dogs without signs of prostatic disease, of a wide range of ages and different body parameters, to then challenge the model with a group of dogs diagnosed with BPH.

There are some works that reported reference values for prostate size based on age and body weight as well as mathematical formulas that predict gland volume using B-mode ultrasound in noncastrated dogs [8, 20, 21]. However, we believe that if the diameter of the abdominal aorta is included as a body criterion in a model to standardize the size and prostate volume of noncastrated dogs, the model can increase the accuracy to differentiate between dogs with normal prostate sizes and those with enlarged prostate sizes. The diameter of the abdominal aorta has previously been used in veterinary medicine to assess abdominal structures size such as the kidney and adrenal gland, since its diameter remains constant over time [22–24]. To date, we have not found a model that simultaneously includes age, the presence or absence of intraparenchymal cysts, and the diameter of the abdominal aorta, to standardize the size and prostate volume of noncastrated dogs. Therefore, the aim of this study was to develop a model that could effectively differentiate between dogs with normal prostate sizes and those with enlarged prostate sizes, by evaluating prostate size and volume using B-mode ultrasound in healthy non-castrated dogs and dogs with BPH, considering factors such as weight, age, aorta diameter (Ao), and the presence of intraparenchymal cysts.

## 2. Materials and Methods

### 2.1. Animals

**Figure 1:**
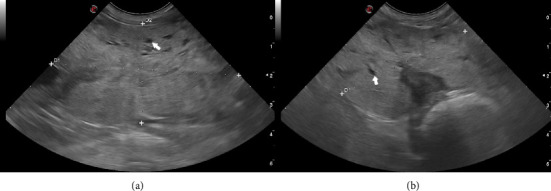
Longitudinal (a) and transverse (b) ultrasound image of a prostate with ultrasound and cytological diagnosis of BPH (between the calipers) of 10-year-old, noncastrated male, mongrel dog. The prostate measures approximately 6.4 × 3.5 × 5.7 cm, with irregular edges, preserved shape, and multiple very small cystic structures (white arrows) in its parenchyma.

The Ethics Committee of the Facultad de Veterinaria de la Universidad de la República approved the present study (CEUA FVET-1554). The study was conducted between January 2022 and October 2023. The owners of the dogs gave their signed consent for the participation of their animals in this study. For this study, two hundred and sixty (*n* = 260) intact male dogs of various breeds were included. The majority were mixed-breed dogs (*n* = 160, 61%), while the most represented pure breeds (*n* = 100, 38%) were Poodle (*n* = 16), Labrador Retriever (*n* = 15), Golden Retriever (*n* = 12), French Bulldog (*n* = 8), Beagle (*n* = 8), Dachshund (*n* = 7), and American Pit Bull Terrier (*n* = 6). Two hundred and thirty-three dogs were included in the healthy control group (healthy group, *n* = 233), with an age range between 1 and 15 years. To be classified in this group, dogs had to exhibit no clinical signs characteristic of prostatic disorders [2, 10] and have normal prostatic morphometry on B-mode ultrasound, as reported by Ruel et al. [8]. These dogs were presented for clinical consultation at the Hospital of the Facultad de Veterinaria–Universidad de la República for various reasons unrelated to genitourinary disorders and for routine health management, including vaccinations and deworming. The other twenty-seven dogs (BPH group, *n* = 27), with an age range between 7 and 15 years, had a presumptive diagnosis of BPH based on clinical signs (hematuria, dripping blood from the urethra, dysuria, and/or tenesmus) and prostate biometry using B-mode ultrasound (Figures 1(a) and 1(b)) [1, 2, 10]. The BPH in this group of animals was confirmed by combining B-mode ultrasound imaging with fine needle aspiration and corresponding cytology [15], as reported by Rodak et al. [25]. Data on breed, body weight (kg), age (years), Ao (cm), length (cm), width (cm), average height (cm), and presence or absence of intraparenchymal cysts were collected for each dog.

### 2.2. B-Mode Ultrasound Evaluation

#### 2.2.1. Prostate

**Figure 2:**
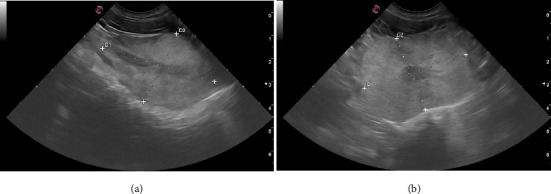
Longitudinal (a) and transverse (b) ultrasound image of normal prostate (between the calipers) of a 2-year-old, noncastrated male, Bernese Mountain Dog.

Measurements of prostate size were obtained by B-mode ultrasound. A single operator (first author, with 15 years of experience in ultrasonography, member of the imaging service of the Facultad de Veterinaria–Universidad de la República) performed ultrasounds using the portable Esaote MyLab OMEGA (Genova, Italy), with a microconvex transducer (mC 3–11) of operating bandwidth between 3 and 11 MHz. The same operator performed two-dimensional ultrasound evaluations and measurements of length, height, and width as in Ruel et al. [8]. The procedure consisted of abdominal examinations to locate the bladder and subsequently identify the prostate. The sagittal position was confirmed when the hypoechoic urethral tract was examined (Figure 2(a)). In sagittal position, the length was measured as the maximum diameter of the gland along the urethral axis, and the height as the maximum diameter perpendicular to the length axis. The transducer was rotated 90° for a cross-sectional image (Figure 2(b)). In this last section, height was measured as the diameter that separates two lobes of the prostate, and width as the maximum diameter perpendicular to the height axis. For each parameter, three consecutive measurements were obtained, with the mean of three values calculated. At the same time, an average was made between height measurements found in the sagittal and transverse slices. From the length, height, and width data, the prostate volume (cm^3^) was determined by the ellipse formula: length × width × height × 0.523 [8, 16]. The presence or absence of intraparenchymal cysts structures was also recorded, as described in the literature [26].

#### 2.2.2. Ao

The Ao immediately cranial to the trifurcation was measured twice and the mean of these values was calculated as in Ruel et al. [8]. The Ao (cm) was measured in the longitudinal plane since it is the least variable plane to measure this artery [22]. Measurements were obtained at the maximum luminal diameter excluding the vessel wall.

### 2.3. Model Construction and Statistical Analysis

Statistical data analysis and visualization were performed using R (Version 4.3.2) and RStudio (Version 2023.12.1 + 402 “Ocean Storm”). Dogs typically experience hyperplastic changes in their prostate glands around the age of 5, and clinical signs of prostate disease begin to appear at around 6 years of age [1, 27]. Therefore, it was decided to categorize dogs into two age ranges: young (≤ 6 years) and adults (> 6 years). Before building the model, a study was conducted to investigate the Pearson correlation between the diameter of the aorta and body weight. Prostate volume was the only quantitative continuous variable that did not follow a normal distribution. Log-transformation (log = natural logarithm) of these data resulted in normal distributions; therefore, the transformed data were used for statistical analysis. The quantitative variables, including length, width, height, and the logarithm of prostate volume, as well as Ao, obtained through ultrasonography in B mode, were analyzed using Student's *t*-test. In contrast, the chi-squared test was employed to compare the qualitative variables, such as cysts and age, between dogs from the healthy group and those from the group with BPH. The construction of a logistic regression model was carried out with the diagnosis of BPH as the dependent variable. We used the Akaike information criterion (AIC) to identify which model best predicted the variables, by appropriately balancing goodness-of-fit and parsimony [28]. The sensitivity, specificity, positive predictive value (PPV), and negative predictive value (NPV) were calculated using the confusion matrix. The accuracy of the model was determined using the confusion matrix, and the receiver operating characteristic (ROC) curve was plotted to show the sensitivity and specificity at various threshold levels. The area under the ROC curve and F1 score were used to measure the overall accuracy of the model. The threshold that provided the maximum sensitivity and specificity was selected as the optimal cutoff value. The results were considered significant at a 95% confidence level.

## 3. Results

### 3.1. Descriptive Analysis of the Population

Table 1 displays a comparison of the continuous and qualitative variables for healthy dogs (healthy group) and dogs with BPH (BPH group). All variables presented differences (*p* < 0.001) between groups except Ao, which was used in the construction of our model as an independent variable of diagnosis of BPH.

In this study, differences in ages were observed between the groups; while 100% of the dogs diagnosed with BPH were over 6 years of age, the healthy group was composed almost half and half between dogs older and younger than 6 years (*p* < 0.001; Table 1). The presence of intraparenchymal cysts increased with the diagnosis of BPH (*p* < 0.001), being observed in 89% of dogs with BPH versus 18% of healthy dogs (Table 1). The prostate variables of length, width, height, and prostate volume (normal and in log scale) were higher for the BPH group compared to healthy group, with 81 cm^3^ of average prostate volume for the BPH group and 22 cm^3^ of average volume for the healthy group (*p* < 0.001).

**Figure 3:**
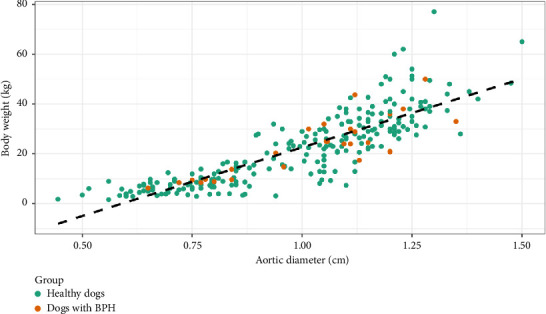
Correlation between body weight (kg) and aorta diameter (cm) in the group of dogs studied. Green circle: healthy dogs; red circle: dogs with BPH.

A high and positive correlation was observed for both groups of dogs (healthy and BPH dogs) between the increase in body weight and the Ao (Person correlation = 0.85, 95% CI [0.82, 0.88], *p* < 0.01) (Figure 3), not showing differences in the correlation between both groups (*p* > 0.05). The presence of prostatic disorder (BPH) did not condition the Ao, the latter being a valid independent parameter for the characterization of the size of the animal in the predictive model for BPH diagnosis (Figure 3).

### 3.2. Development of a Predictive Model and Its Precision to Assess Prostatic Dimensions in Dogs Diagnosed With BPH

A logistic regression model was applied with BPH diagnosis as the dependent variable (variable dichotomous; e.g., absent = healthy or present = BPH), where the predictions of total or partial inclusion of all variables (independent variables; discrete [intraparenchymal cysts; Age] or continuous [Ao; log prostate volume]) were verified based on goodness-of-fit and the AIC statistical criteria. The model with the lower AIC score was considered to be the better one [28] (Supporting 1). The optimal model (Model 1 and its equation) included as predictor variables in the analysis the diameter of the aorta, the logarithm of the prostate volume, the presence (Value 1 is entered) or absence (Value 0 is entered) of intraparenchymal cysts, and the age of the dog older (Value 1 is entered) or younger (Value 0 is entered) than 6 years (Table 2). This suggests that higher values of log prostate volume are associated with a greater likelihood of the BPH diagnostic.

The logistic regression equation is the following:

(1) $$\begin{array}{c} \ln\left[\frac{P\left(\text{Diagnostic}=\text{BPH}\right)}{1-P\left(\text{Diagnostic}=\text{BPH}\right)}\right]=\alpha +\beta_{1}\left(\text{Aorta Diameter}\left[\text{cm}\right]\right)+\beta_{2}\left(\mathrm{Log}\text{ prostate volume}\right)+\beta_{3}\left({\text{Cysts}}_{\text{yes}}\right)+\beta_{4}\left({\text{Age}}_{>6\text{years}}\right), \end{array}$$

(2) $$\begin{array}{c} \alpha =-45.66;\beta_{1}=-28.29;\beta_{2}=13.27;\beta_{3}=3.6;\beta_{4}=21.48, \end{array}$$

where *p* = Pr {*Y* = 1} and Logit (*p*) = ln (*p*/1 − *p*) and *p* can be calculated by taking the inverse of the Logit (*p*) (*p* = _inv_Logit/1 + _inv_Logit), where p represents the probability of the presence of BPH when the findings variables are identified (Supporting 2; Supporting Spreadsheet).

It is very important to mention that our model establishes values greater than a cutoff of 0.22% as indicative of a positive diagnosis of BPH. To illustrate the use of the logistic regression model to estimate the probability of BPH, we present two hypothetical cases (Supporting 3). Firstly, a male dog under 6 years of age was considered, with an Ao of 1.05 cm, a prostate volume of 15 cm^3^, and no presence of prostatic cysts (Supporting 3, Case 1). The probability of BPH for this finding using our model was 2.2e^−16^% (95% CI = −1.25e^−12^% to 1.25e^−12^%). The model indicates a very low probability of BPH. Conversely, in the second case, we evaluate a 10-year-old male dog with an Ao of 1.1 cm, a prostate volume of 65.4 cm^3^, and multiple cystic structures (Supporting 3, Case 2). The model estimated its probability of BPH equal to 0.97% (95% CI = 0.91% to 1.04%), indicative of a high probability of BPH. This last case represents a straightforward case of BPH where a variable increases the probability of diagnostic using our model.

Percent model accuracy measures the accuracy of a model in predicting outcomes. In the classification table (Table 2), considering all the dependent variables included in the model, the number of normal dogs that were observed and predicted was equal to 231 while 24 dogs with pathological findings were observed and predicted as pathological. Therefore, the accuracy rate was calculated at 98.1% (231 + 24/260). On the other hand, the misclassification rate (percentage of wrongly predicted observation) was 1.92% and was obtained as 2 (false positive) +3 (false negative) divided by 260.

**Figure 4:**
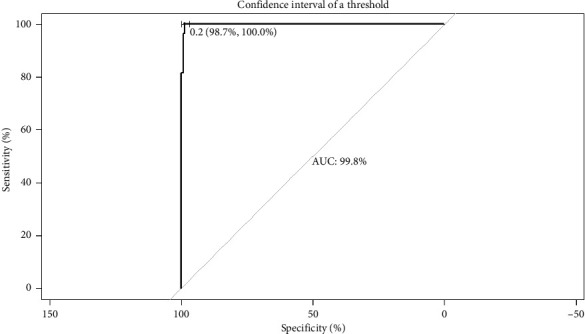
Area under the curve of the receiver operated characteristics (AUC-ROC) curve for the BPH model incorporating diameter of the aorta, the logarithm of the prostate volume, the presence or absence of intraparenchymal cysts, and the age of the dog as covariate. The AUC is shown in the figure with 95% confidence interval between brackets and cutoff point used.

The plot of sensitivity versus 1 − specificity is called the ROC curve and the area under the curve (AUC) is an effective measure of accuracy.  Figure 4 shows the ROC curve of the model, indicating the cutoff point used and the AUC value. For the calculation of model sensitivity (percentage of correctly predicted events), specificity (percentage of nonoccurrences that are correctly predicted), false positive rate (FPR) (percentage of nonoccurrences that are incorrectly predicted as events), and false negative rate (FNR) (percentage of events that are incorrectly predicted), a cutoff value was set at 0.22. On calculation, the sensitivity of the model is at 88.9% and the specificity is at 99.1%. At the same time, the results obtained from FPR and FNR were higher than desired (0.86% and 11.1%, respectively).

## 4. Discussion

The optimal model (Model 1) was determined to be the one that incorporated variables by B-mode ultrasound such as the logarithm of prostate volume, Ao, and the presence or absence of intraparenchymal cysts, along with the age of the dog. According to Markowetz [29], for a clinical prediction model to be successful, it should meet certain requirements, such as addressing a clear clinical decision point, generating parameters that help decision making, using data that are available in routine practice, and improving preexisting tools, among others. Our model, based on B-mode ultrasound images, demonstrated a high level of accuracy in discriminating between prostate sizes of healthy noncastrated male dogs and prostates with BPH (Figure 4). Our results show that the combination of a logistic regression model and ultrasound evaluation would contribute to discriminate between normal and BPH dogs. Moreover, the model would avoid the diagnosis of false negative cases in comparison with the single observation of the ultrasound evaluation without an interpretation model of the variables. The adoption of this model will allow clinical decisions to be made based on objective numerical data, using a fast, accessible, and noninvasive technique such as B-mode ultrasound.

As expected, our results show that the presence/absence of intraparenchymal cysts, length, width, height, and logarithm volume of the dog prostate estimated by B-mode ultrasound, as well as age, presented significant differences between healthy noncastrated dogs and dogs with BPH. The differences between the two groups (healthy and BPH) may be attributed to the fact that the prostate of intact male dogs gradually increases in weight and size with age, with histological evidence of hyperplasia observed in approximately 90% of dogs over 8 years old [6]. Despite this, clinical signs may not manifest until prostate enlargement is sufficient to produce them [1], so some dogs with BPH may be asymptomatic [30]. In this sense, Polisca et al. [3] recommend B-mode ultrasound for prostate evaluation in intact male dogs aged 6–7 years to detect prostate disorders early. Ultrasound reveals prostatic changes, including increased length, width, height, and volume logarithm, with age [8, 20]. These changes predispose dogs to prostate disorders, with BPH being the most common [2–4]. Our results confirmed an association between prostate size increase and higher BPH probability. This model provides a fast, accessible, noninvasive tool for detecting BPH in intact males over 6 years old before clinical signs appear, allowing early medical intervention. On the other hand, prostatic hyperplasia initially manifests as glandular and later becomes cystic, forming fluid-filled structures due to canaliculi obstruction [1, 10, 12]. In this study, intraparenchymal cysts were associated with BPH, though 18% of healthy dogs had cysts, and 11% with BPH did not. Since B-mode ultrasound easily detects cysts, we included their presence as a predictor for BPH, as cyst formation correlates with increased prostate dimensions and volume, improving prostate enlargement diagnosis.

In this study, the only variable in which no differences were seen between healthy and BPH groups was the Ao. This result was expected since in previous work in dogs it was observed that the Ao is not influenced by sex or age [23], nor by body weight [22]. In turn, it has been seen in humans that the aorta remains constant even with intravascular volume loss and that it does not vary before or after intravenous fluid administration [31]. This is the reason why we decided to include the Ao in our model as an indicator of the body size. For a measurement method to be adopted and validated, it must meet certain performance characteristics, an important one being that it is reproducible. In this sense, the diameter of the aorta is an easy measurement to determine during a B-mode ultrasound study. It has been seen that the maximum luminal Ao is more consistent in the longitudinal plane, being easily measured by sonographers with different levels of experience [22]. For this reason, we studied the prediction of the body weight of dogs through the ultrasound measurement of the Ao. We were able to observe, as in previous studies [22, 23] the positive relationship between the increase in body weight and the Ao. At the same time, the presence of BPH did not condition the Ao, the latter being a valid independent parameter for the characterization of the size of the animal in the predictive model for BPH diagnosis.

One of the biggest challenges when measuring the prostate by B-mode ultrasound is that it is an operator-dependent method. In this sense, Leroy et al. [32] studying the reproducibility and repeatability between observers for prostate measurements in healthy Beagle dogs reported differences between three observers for most of the measurements, probably due to the difference in experience of each operator. Despite this, in this same study, a lower coefficient of variation between observers was obtained for the height than the length in the longitudinal plane and for the measurement of width in the transversal plane, with the measurement of width being the one with the lowest interobserver coefficient of variation [32]. However, other authors mention that they did not observe differences between the repeated measurements of length and height of the prostate, but they did find significant differences between the repeated measurements of the width [9]. In turn, prostatic enlargement is not uniform, with greater growth in length than in other dimensions [33]. This could explain the difficulty in measuring prostate length in the longitudinal plane of dogs with BPH, as the caudal edge overlapped with the pelvic bone, possibly leading to underestimation. To mitigate this, B-mode ultrasonographies were performed with a distended bladder, facilitating prostate evaluation [26]. Measurements were taken three times and averaged to reduce error. On the other hand, prostate involution after castration, as estimated by B-mode ultrasound, varies by parameter (length, width, or height) between BPH and healthy dogs, suggesting androgen sensitivity differs across gland regions [16]. However, prostate volume decreases similarly in both groups, indicating it may be the best variable for estimating size via ultrasound. Measuring a single parameter could misrepresent prostate size, whereas volume, integrating height, length, and width, better reflects enlargement and its changes for castration, age, and body size [34]. Thus, our model prioritizes prostate volume, expressed logarithmically for normal data distribution.

This study has some limitations. In our study, we were unable to determine the biomarker canine prostate–specific arginine esterase (CPSE) for the diagnosis of BPH in dogs. Although we know that being able to determine CPSE could have helped to have an objective marker on the health status of the dogs in the control group [35, 36], for logistical and economic reasons, it was unfortunately not determined. However, it is also important to indicate that the control group of dogs was not only evaluated by B-mode ultrasound, but also for the absence of clinical signs associated with prostatic disorders. Although the presence of prostate disorders cannot be entirely ruled out in this group of animals, as no cytological or histological analyses were performed due to the invasiveness of the procedure and the reluctance of the guardians, we consider it unlikely given the inclusion criteria. Conversely, cytological confirmation of BPH was required for dogs with clinical suspicion and compatible ultrasound findings, as per Rodak et al. [25], justified by clinical presentation and guardian consent. Additionally, BPH in dogs under 6 years cannot be excluded, as cytology was only performed in symptomatic cases. While prostate enlargement typically occurs after six years [1], prostate disorders are most common between 8 and 10 years [3, 4]. Thus, we consider that categorizing age as ≤ 6 and > 6 years is appropriate, as the likelihood of prostate enlargement or disease before 6 years is minimal.

Another possible limitation of this model is that for its construction, only the ellipse formula was used to calculate prostate volume. Although the prostate is not a perfect ellipse, the formula is easy to apply and has been used in other works in veterinary medicine, both for its calculation from B-mode ultrasound images [8, 16, 21] and by CT [34]. In this sense, Kamolpatana et al. [21] observed that dog prostate volume values derived from the ellipse formula overestimated the real volume determined by water displacement, but these differences were small. In turn, the volume obtained by the ellipse formula has been used successfully in the diagnosis of BPH, as well as in the follow-up of dogs with BPH after testosterone suppression through castration [16]. Atalan et al. [9], comparing ultrasound measurements of prostates and their correlation in cadavers, discuss that the formula derived from their study in dogs does not differ greatly from the ellipse formula. In any case, we consider it interesting in the future to verify and compare the sensitivity, specificity, and accuracy of the model using the formula for estimating prostate volume by B-mode ultrasound described by Atalan et al. [9] as well as that of other authors [21] as an alternative to the ellipse formula.

Finally, our research highlights the significance of the operator's role in conducting ultrasound assessments. Additional studies involving more operators utilizing a comparable methodology to obtain and measure B-mode ultrasound images would further validate the proposed model's applicability, despite the potential interobserver variability. The proposed model emphasizes the importance of B-mode ultrasound in making diagnostic decisions.

## 5. Conclusion

Our study has culminated in the creation of a model that is highly effective in differentiating healthy, noncastrated dogs from those with BPH with a great degree of accuracy, sensitivity, and specificity. The model employs prostatic volume estimates obtained via B-mode ultrasound to make these distinctions.

## Figures and Tables

**Table 1:** Population characteristics of the variables aorta diameter (cm), length (cm), width (cm), height (cm), prostate volume (cm^3^), cysts (No; Yes), age (< 6; > 6 years), and logarithm of the prostate volume according to healthy dogs (healthy group) and dogs with BPH (BPH group).

Diagnostic				
Variable	*N*	Healthy group, *N* = 233^1^	BPH group, *N* = 27^1^	*p* value^2^
Aorta diameter (cm)	260	0.98 (0.22)	1.02 (0.19)	0.34
Length (cm)	260	3.58 (0.83)	5.86 (1.13)	**< 0.001**
Width (cm)	260	3.53 (0.81)	5.27 (1.03)	**< 0.001**
Height (cm)	260	2.92 (0.73)	4.60 (1.03)	**< 0.001**
Prostate volume (cm^3^)	260	22 (15)	81 (41)	**< 0.001**
Cysts	260			**< 0.001**
No		191 (82%)	3 (11%)	
Yes		42 (18%)	24 (89%)	
Age (years)	260			**< 0.001**
< 6 years		115 (49%)	0 (0%)	
> 6 years		118 (51%)	27 (100%)	
Log vol	260	2.59 (0.49)	2.87 (0.71)	**< 0.001**

*Note:* Log vol: logarithm of prostate volume. Values in bold indicate *p* < 0.05.

^1^Mean (SD); *n* (%).

^2^Welch two-sample *t*-test: Pearson's chi-squared test.

**Table 2:** Population characteristics of the variables aorta diameter (cm), length (cm), width (cm), height (cm), cysts (No; Yes), age (< 6; > 6 years), and logarithm of the prostate volume according to healthy dogs (healthy group) and dogs with BPH (BPH group).

Variable	*N*	Log (OR)^1^	95% CI	*p* value
Aorta diameter (cm)	260	−28	−53, −14	**0.002**
Log vol	260	13	7.3, 24	**0.001**
Cysts	260			
No		—	—	
Yes		3.6	0.67, 8.6	
Age (years)	260			**0.057**
< 6 years		—	—	
> 6 years		21	−242, NA	> 0.9

*Note:* OR: odds ratio (logistic regression model); Log vol: logarithm of prostate volume. Values in bold indicate *p* < 0.05.

Abbreviation: CI, confidence interval.

## Data Availability

The corresponding author can provide the data that support the findings of this study upon reasonable request.

## Supporting Information

Supporting Information 1 Supporting 1. Comparative study of the application of logistic regression models for the diagnosis of BPH (dependent variable) in canines for the total or partial inclusion of predictor variables (independent variables). Verification was performed based on goodness-of-fit and Akaike's information criterion (AIC) statistical criteria. Supporting 2. Elucidation of the logistic regression equation and its constituent elements in simplified terms. Supporting 3. The probability distribution of benign prostatic hyperplasia (BPH) across different prostatic volumes (cm^3^) in the ultrasound study of 260 dogs is illustrated in the figure. The size of each dot corresponds to the individual aorta diameter (cm), while the curve line (solid or broken) and color indicate the presence or absence of cysts and the age range of the dog, respectively. The figure displays an example of the probability for case 1 (male dog weighing 20 kg, under 6 years of age, with an aorta diameter of 1.05 cm, a prostate volume of 15 cm^3^, and no presence of prostatic cysts) and case 2 (10-year-old male dog with an aorta diameter of 1.1 cm, a prostate volume of 65.4 cm^3^, and multiple cystic structures).

Supporting Information 2 Supporting Spreadsheet. A mathematical model has been implemented in a spreadsheet to estimate the probability of a BPH diagnosis. This tool allows for individualized risk assessment based on multiple clinical parameters.
