# Supplementary material for: Development of a Model for Predicting Enlarged Prostate Size in Noncastrated Dogs Through B-Mode Ultrasound
Source: Vet Med Int. 2025 Jul 21;2025:9593213. doi: 10.1155/vmi/9593213 (PMC12303645; doi:10.1155/vmi/9593213)
Supplement: Supporting Information 1 — Supporting 1. Comparative study of the application of logistic regression models for the diagnosis of BPH (dependent variable) in canines for the total or partial inclusion of predictor variables (independent variables). Verification was performed based on goodness-of-fit and Akaike's information criterion (AIC) statistical criteria. Supporting 2. Elucidation of the logistic regression equation and its constituent elements in simplified terms. Supporting 3. The probability distribution of benign prostatic hyperplasia (BPH) across different prostatic volumes (cm3) in the ultrasound study of 260 dogs is illustrated in the figure. The size of each dot corresponds to the individual aorta diameter (cm), while the curve line (solid or broken) and color indicate the presence or absence of cysts and the age range of the dog, respectively. The figure displays an example of the probability for case 1 (male dog weighing 20 kg, under 6 years of age, with an aorta diameter of 1.05 cm, a prostate volume of 15 cm3, and no presence of prostatic cysts) and case 2 (10-year-old male dog with an aorta diameter of 1.1 cm, a prostate volume of 65.4 cm3, and multiple cystic structures). [file 9593213.f1.docx]

**SUPPLEMENT**

Supplement 1. Comparative study of the application of logistic regression models for the diagnosis of BPH (dependent variable) in canines for the total or partial inclusion of predictor variables (independent variables). Verification was performed based on goodness-of-fit and Akaike's Information Criterion (AIC) statistical criteria.

|  | **model 1** | **model 2** | **model 3** | **model 4** | **model 5** | **model 6** | **model 7** |
| --- | --- | --- | --- | --- | --- | --- | --- |
| **(Intercept)** | -45.66 | -17.52*** | -41.80 | -16.98*** | -33.46 | -22.68 | -2.16*** |
|  | (2885.16) | (4.49) | (2895.47) | (4.31) | (2147.96) | (1529.88) | (0.20) |
| **Aorta diameter (cm)** | -28.29** | -23.02*** | -25.05*** | -23.21*** |  | 1.34 |  |
|  | (9.33) | (6.50) | (7.18) | (5.74) |  | (1.11) |  |
| **Log (Prostate volume (cm3))** | 13.27** | 10.11*** | 12.33*** | 10.56*** | 3.18*** |  |  |
|  | (4.06) | (2.58) | (3.33) | (2.39) | (0.67) |  |  |
| **Intraparenchymal cysts (Presence)** | 3.60+ | 2.70+ |  |  | 2.39** | 3.06*** |  |
|  | (1.89) | (1.43) |  |  | (0.79) | (0.65) |  |
| **Age (> 6 year)** | 21.48 |  | 20.48 |  | 18.94 | 17.98 |  |
|  | (2885.15) |  | (2895.46) |  | (2147.96) | (1529.88) |  |
| *Num.Obs.(n)* | 260 | 260 | 260 | 260 | 260 | 260 | 260 |
| *AIC* | 28.9 | 35.4 | 33.3 | 38.4 | 65.8 | 110.3 | 175.4 |
| *BIC* | 46.7 | 49.7 | 47.5 | 49.1 | 80.1 | 124.5 | 179.0 |
| *Log-.Lik.* | -9.465 | -13.711 | -12.625 | -16.219 | -28.911 | -51.137 | -86.698 |
| *F* | 2.762 | 5.498 | 4.562 | 9.746 | 9.919 | 7.600 |  |
| *RMSE* | 0.11 | 0.13 | 0.12 | 0.14 | 0.18 | 0.25 | 0.31 |
| + p < 0.1, * p < 0.05, ** p < 0.01, *** p < 0.001; The selected optimal model (model 1) is highlighted in gray. *Num.Obs.: number observed; AIC: Akaike Information Criterion; BIC: Bayecian Information Criterion; Log-Lik.: logarithm -Likelihood Computation; F: Fisher information; RMSE: Root Mean Squared Error; Data are reporter with coefficient (standard error)* | | | | | | | |

Supplement 2. **Elucidation of the logistic regression equation and its constituent elements in simplified terms**

$$\begin{matrix} \ln\left[ \frac{P\left( Diagnostic=BPH \right)}{1-P\left( Diagnostic=BPH \right)} \right] & =\alpha+\beta_{1}\left( Aorta Diameter [cm] \right)+\beta_{2}\left( Log prostate volume \right)+ \\ & \beta_{3}\left( \mathrm{Cysts}_{\mathrm{yes}} \right)+\beta_{4}\left( Age{}_{>6years} \right) \end{matrix}$$

$$\alpha= - 45.66; \beta_{1}=- 28.29; \beta_{2}= 13.27; \beta_{3}= 3.6;\beta_{4}= 21.48$$

**Objective:**

The objective of this equation is to estimate the probability of a diagnosis of Benign Prostatic Hyperplasia (BPH) based on multiple clinical parameters.

**1. The Core Equation:**

- **ln[P(Diagnostic=BPH)/(1-P(Diagnostic=BPH)**: This is the logit transformation of the probability of having BPH. "P(Diagnostic=BPH)" represents the probability that a patient has BPH. The logit transformation is used because logistic regression models the relationship between the predictors and the log-odds of the outcome, not the probability directly. This transformation allows us to use a linear equation to model a probability that is bounded between 0 and 1.
- **α**: This is the intercept. It's the log-odds of BPH when all predictor variables are zero. In this case, α = -45.66.
- **β₁, β₂, β₃, β₄**: These are the coefficients for each predictor variable. They represent the change in the log-odds of BPH for a one-unit change in the corresponding predictor, holding all other variables constant.
  - β₁ = -28.29 (Aorta Diameter): A one-unit increase in aorta diameter (cm) is associated with a decrease in the log-odds of BPH by 28.29, all else being equal. The negative sign indicates an inverse relationship.
  - β₂ = 13.27 (Log prostate volume): A one-unit increase in the log of prostate volume is associated with an increase in the log-odds of BPH by 13.27, all else being equal.
  - β₃ = 3.6 (Cysts_yes): This is a binary variable. If cysts are present (Cysts_yes = 1), the log-odds of BPH are increased by 3.6 compared to when cysts are absent (Cysts_yes = 0).
  - β₄ = 21.48 (Age_(>6years)): This is also likely a binary variable. If the patient is older than 6 years, the log-odds of BPH are increased by 21.48 compared to if the patient is 6 years or younger. (It's unusual to consider BPH in such young patients, so the meaning of this variable should be carefully considered.)
- **(Aorta Diameter [cm]), (Log prostate volume), (Cysts_yes), (Age_(>6years))**: These are the predictor variables. Their values are plugged into the equation to calculate the log-odds of BPH.

**2. Calculating the Probability (p): p = invLogit / 1 + invLogit**

- **invLogit**: This is the inverse of the logit transformation. It's calculated as exp(α + β₁ (Aorta Diameter) + β₂ (Log prostate volume) + β₃ (Cysts_yes) + β₄ (Age_(>6years))) or, more simply, as e raised to the power of the entire right side of the main equation.
- **p**: Once you have the invLogit, you can calculate the probability of BPH using the formula p = invLogit / (1 + invLogit). This converts the log-odds back into a probability value between 0 and 1.

**In summary:** You plug the patient's aorta diameter, log of prostate volume, cyst presence (yes/no), and age (older than 6 years/not) into the equation. This gives you the log-odds of BPH. You then use the invLogit and the final formula to convert the log-odds into a probability (p) of having BPH. This probability can then be used to help in diagnosis or risk assessment. (See Spreadsheet).


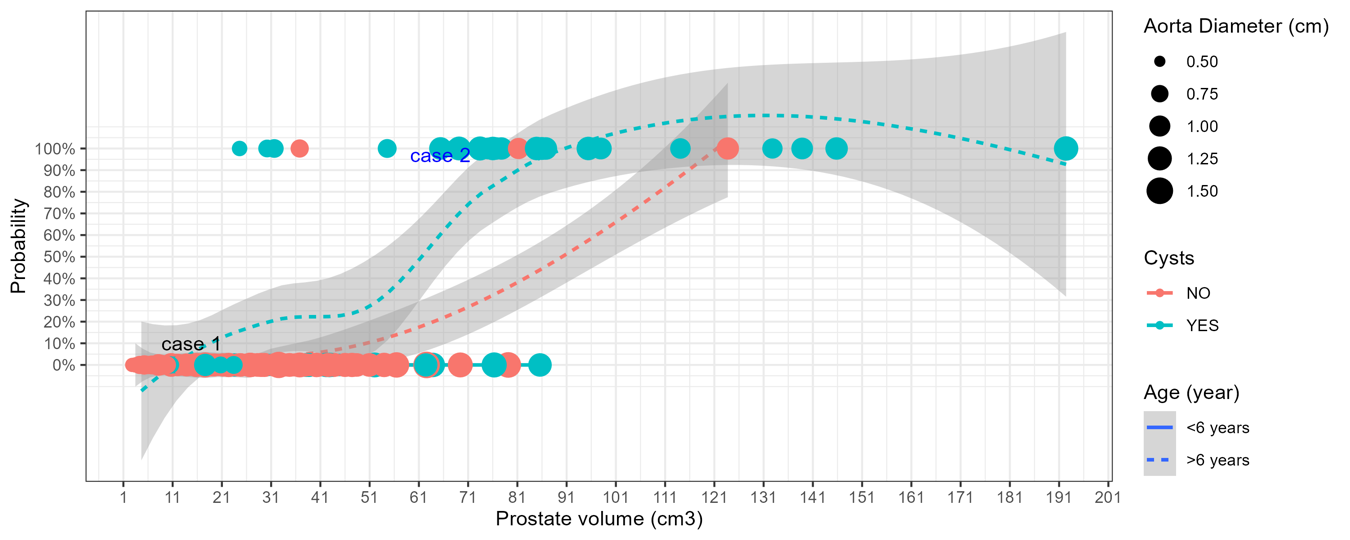


Supplement 3: The probability distribution of Benign Prostatic Hyperplasia (BPH) across different prostatic volumes (cm^3^) in the ultrasound study of 260 dogs is illustrated in the figure. The size of each dot corresponds to the individual aorta diameter (cm), while the curve line (solid or broken) and color indicate the presence or absence of cysts and the age range of the dog, respectively. The figure displays an example of the probability for cases 1 (male dog under 6 years of age, with an aorta diameter of 1.05 cm, a prostate volume of 15 cm³, and no presence of prostatic cysts) and case 2 (10-year-old male dog with an aorta diameter of 1.1 cm, a prostate volume of 65.4 cm³, and multiple cystic structures).
